# *CXCL12* rs1801157 Polymorphism Is Associated with Antiatherogenic Lipoprotein Subfraction Profile Independent of Coronary Artery Disease Risk in a Turkish Population: A Case–Control Study

**DOI:** 10.3390/jcm15114206

**Published:** 2026-05-29

**Authors:** İnci Deniz, Ayça Türer Cabbar, Fatma Tuba Akdeniz, Turgay İsbir, Seda Güleç Yılmaz

**Affiliations:** 1 Department of Molecular Medicine, Institute of Health Sciences, Yeditepe University, Istanbul 34755, Turkey; turgay.isbir@yeditepe.edu.tr (T.İ.); seda.gulec@yeditepe.edu.tr (S.G.Y.); 2 Yağmur Sönmez Microbiology R&D Laboratory, Faculty of Pharmacy, Yeditepe University, Istanbul 34755, Turkey; 3 Department of Cardiology, Faculty of Medicine, Yeditepe University, Istanbul 34755, Turkey; ayca.turer@yeditepe.edu.tr; 4 Department of Genetics and Bioengineering, Faculty of Engineering and Natural Sciences, Istanbul Okan University, Istanbul 34959, Turkey; fatma.akdeniz@okan.edu.tr; 5 Department of Medical Biology, Faculty of Medicine, Yeditepe University, Istanbul 34755, Turkey

**Keywords:** *CXCL12*, SDF-1, rs1801157, coronary artery disease, lipoprotein subfractions, HDL, polymorphism, Turkish population

## Abstract

**Background/Objectives**: Cardiovascular diseases remain a leading cause of global mortality. The C-X-C motif chemokine ligand 12 (*CXCL12)* gene has been implicated in atherosclerosis; however, its relationship with lipoprotein subfraction profiles remains unclear. The primary objective of this study was to investigate the association between the *CXCL12* rs1801157 C>T single nucleotide polymorphism (SNP) and coronary artery disease (CAD) risk in a Turkish population. The secondary objective was to evaluate the relationship between this polymorphism and LDL and HDL lipoprotein subfraction profiles. **Methods**: This case–control study included 139 patients with angiographically confirmed CAD and 125 healthy controls. Genotyping was performed using TaqMan real-time polymerase chain reaction (PCR). Low-density lipoprotein (LDL) and high-density lipoprotein (HDL) subfractions were analyzed using the Lipoprint^®^ polyacrylamide gel electrophoresis system. Multivariable logistic and linear regression analyses were performed, adjusting for age, sex, body mass index (BMI), and major cardiovascular risk factors. **Results:** No significant differences in rs1801157 genotype or allele distributions were observed between groups (overall χ^2^ = 0.459, *p* = 0.796). Logistic regression confirmed that the polymorphism was not an independent predictor of CAD risk (CT: OR = 1.396, *p* = 0.409; TT: OR = 1.458, *p* = 0.694). HDL-C was an independent protective factor (OR = 0.952, 95% CI: 0.910–0.996; *p* = 0.029). Notably, TT homozygous carriers exhibited significantly higher large HDL (*p* = 0.018) and intermediate HDL (*p* < 0.001) subfraction levels and markedly lower small LDL concentrations (*p* < 0.001). Multivariable linear regression confirmed these associations were independent of age, sex, and BMI. **Conclusions:** The CXCL12 rs1801157 variant does not directly influence CAD susceptibility but modulates lipoprotein quality by promoting larger HDL subfractions and reducing atherogenic small LDL particles, suggesting an indirect cardioprotective role through lipid metabolism.

## 1. Introduction

Coronary artery disease (CAD) remains a leading cause of cardiovascular mortality worldwide, primarily resulting from atherosclerotic narrowing of the coronary arteries [1,2]. Its pathogenesis is driven by a complex interplay of lifestyle, environmental, and genetic factors, while the increasing prevalence of obesity and diabetes has further amplified atherosclerotic risk across populations [3,4]. Family-based and twin studies have demonstrated a substantial heritable component in CAD, with genetic factors estimated to account for approximately 40–60% of disease susceptibility [5,6]. In recent years, large-scale genome-wide association studies have identified hundreds of coronary artery disease susceptibility loci, confirming its highly polygenic architecture and revealing complex biological pathways involved in atherosclerosis [6,7]. Despite these advances, the biological relevance and functional impact of many CAD-associated loci remain incompletely understood, particularly regarding their influence on lipid metabolism and lipoprotein functionality [6,7,8].

Atherosclerosis is a chronic inflammatory disease initiated by endothelial dysfunction and characterized by intimal lipid accumulation, foam cell formation, vascular smooth muscle cell proliferation, and plaque instability [9,10].

Among the key mediators of these processes, chemokines play a central role in regulating leukocyte recruitment and vascular inflammation. C-X-C motif chemokine ligand 12 (CXCL12), also known as stromal cell-derived factor-1 (SDF-1), is encoded by the CXCL12 gene on chromosome 10q11.21. CXCL12 exerts its biological effects primarily through binding to the C-X-C motif chemokine receptor 4 (CXCR4), thereby contributing to inflammatory cell recruitment and atherosclerotic progression [9,11,12].

Among CAD susceptibility loci identified by genome-wide association studies (GWAS), the 10q11 region has been repeatedly implicated, highlighting the potential involvement of the CXCL12 genomic region in atherosclerotic disease biology. However, the functional relevance of the CXCL12 rs1801157 C>T polymorphism remains controversial across different ethnic populations. While some studies have reported associations with cardiovascular and lipid-related phenotypes, others have failed to confirm these findings, suggesting possible population-specific effects and biological heterogeneity [6,8,13]. In this context, the rs1801157 polymorphism was selected as a candidate variant due to its reported regulatory potential and inconsistent associations with CAD-related traits.

High-density lipoprotein (HDL) exerts cardioprotective effects primarily through reverse cholesterol transport (RCT), a process that facilitates cholesterol efflux from peripheral tissues and macrophage foam cells to the liver for excretion. Epidemiological and mechanistic studies have demonstrated an inverse relationship between HDL functionality and CAD risk; however, HDL functionality and cholesterol efflux capacity appear to be more clinically relevant than circulating HDL-C levels alone [14,15,16]. HDL is a heterogeneous class of lipoproteins, and larger HDL particles exhibit greater efficiency in cholesterol efflux as well as stronger antioxidant and anti-inflammatory properties compared with smaller subfractions. Therefore, HDL particle composition and functionality may provide more accurate information for cardiovascular risk assessment [16]. Emerging evidence suggests that CXCL12/CXCR4 signaling may contribute to lipid metabolism and cholesterol homeostasis through modulation of ATP-binding cassette transporter A1 (ABCA1)-mediated cholesterol efflux pathways. Since ABCA1 plays a critical role in nascent HDL formation and reverse cholesterol transport, genetic variations affecting CXCL12 signaling may indirectly influence lipoprotein metabolism and atherosclerotic risk [17,18,19,20].

To our knowledge, no study has evaluated the association between the *CXCL12* rs1801157 C>T single nucleotide polymorphism (SNP) and detailed lipoprotein subfraction profiles in a Turkish population. Therefore, this study aimed to (i) investigate whether rs1801157 is associated with CAD susceptibility in a Turkish cohort and (ii) assess its relationship with LDL and HDL subfraction distribution, to determine whether this variant influences cardiovascular risk through modulation of lipoprotein quality.

## 2. Materials and Methods

### 2.1. Study Population and Clinical Procedures

This case–control study enrolled 139 patients with angiographically confirmed CAD and 125 healthy controls at the Department of Cardiology, Yeditepe University, Istanbul, Turkey. Inclusion criteria were: age ≥18 years and non-pregnant status. Exclusion criteria included prior coronary artery bypass surgery, presence of acute coronary syndrome at the time of evaluation, and any condition precluding coronary angiography.

CAD was defined as ≥50% stenosis in at least one major coronary artery on angiography; lesion severity was further quantified by the Synergy Between PCI With Taxus and Cardiac Surgery score (SYNTAX score). Cardiovascular risk factors including hypertension, diabetes mellitus (DM), hyperlipidemia, family history of CAD, and smoking status were recorded. All procedures adhered to the Declaration of Helsinki. Ethical approval was granted by the Yeditepe University Medical Faculty Ethics Committee (No. 2022-KAEK-2409, Decision No. 1607, 12 May 2022). Detailed information about the study was given to the participants and written informed consent was obtained from all of them before their inclusion.

### 2.2. Genomic DNA İsolation and Quality Assessment

**Figure 1:**
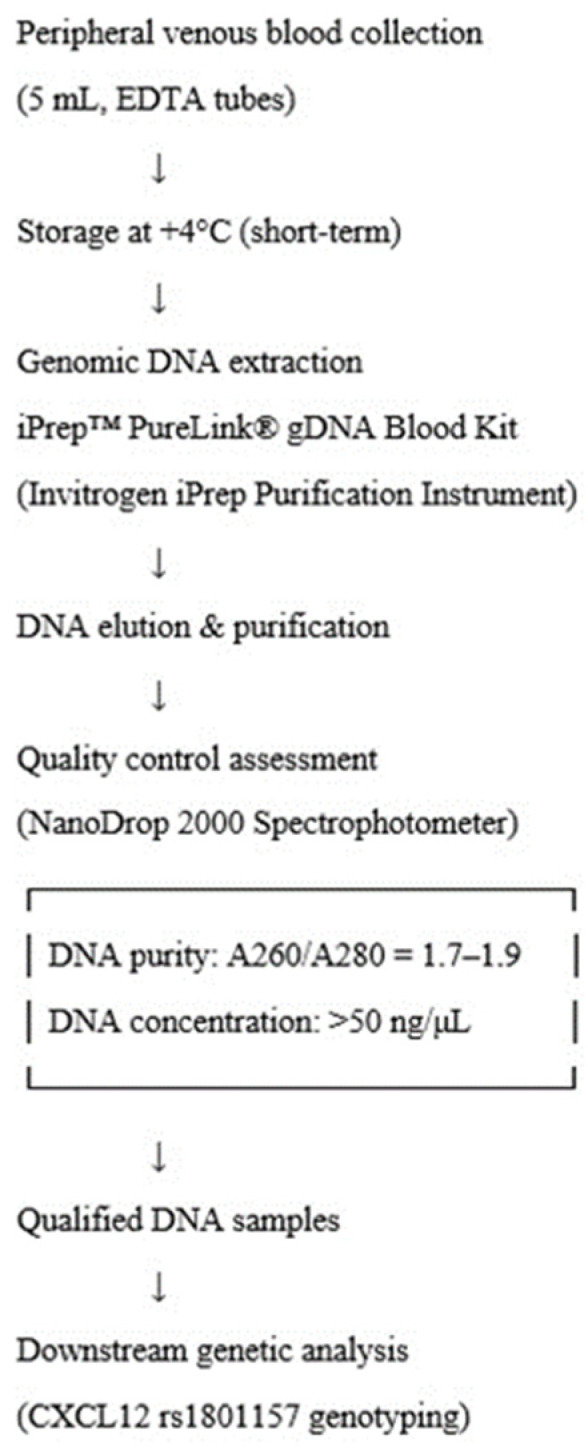
Workflow of genomic DNA isolation and quality assessment in study participants.

Peripheral venous blood (5 mL EDTA) was collected and stored at +4 °C. Genomic DNA was extracted using the iPrep™ PureLink^®^ gDNA Blood Kit on the Invitrogen iPrep Purification Instrument (Life Technologies, Carlsbad, CA, USA). DNA purity (A260/A280 ratio: 1.7–1.9) and concentration (>50 ng/μL) were confirmed by NanoDrop 2000 spectrophotometry (Thermo Scientific, Waltham, MA, USA) (Figure 1).

### 2.3. Genotyping

The C-X-C motif chemokine ligand 12 (CXCL12) rs1801157 C>T single nucleotide polymorphism (SNP) was selected based on its known involvement in cardiovascular and inflammatory processes [21]. CXCL12 is a chemokine that participates in leukocyte trafficking and vascular inflammation through interaction with the CXCR4 receptor, processes that are central to atherogenesis [21]. The CXCL12 gene is located on chromosome 10q11, a genomic region that has been repeatedly implicated in susceptibility to coronary artery disease in large-scale genome-wide association studies [22]. The rs1801157 variant is located within the 3′ untranslated region of CXCL12 and has been frequently investigated in relation to cardiovascular traits and lipid-related phenotypes, although results across studies remain inconsistent [8]. Population-based allele frequency data from large-scale genomic databases (e.g., gnomAD) indicate that the rs1801157 variant has a relatively common minor allele frequency across different populations, including European and Asian ancestry groups, supporting its suitability for genetic association studies [23]. Therefore, rs1801157 was selected to evaluate its potential relationship with coronary artery disease risk and lipoprotein subfraction profiles in the present study.

Genotyping for rs1801157 was performed using TaqMan SNP Genotyping Assays on the Applied Biosystems 7500 Fast Real-Time PCR system (Applied Biosystems, Foster City, CA, USA). Reactions (10 μL) contained 5 μL Genotyping Master Mix, 0.25 μL TaqMan assay, 1 μL genomic DNA, and 3.75 μL nuclease-free water. Cycling: 95 °C for 10 min; 40 cycles of 92 °C for 15 s and 60 °C for 60 s. Allelic discrimination (FAM/VIC channels; ROX passive reference) was analyzed using 7500 Fast software and confirmed via Thermo Fisher Cloud.

Primers: forward 5′-GCTGCCCTCCCAGAAGAGGCAGACC-3′;

reverse 5′-GGCTCCCATGTGGATGGAGAAGGGG-3′.

### 2.4. Lipoprotein Subfraction Analysis

LDL and HDL subfractions were quantified using the Lipoprint^®^ Lipoprotein Subfraction Testing System (Quantimetrix Corporation, Redondo Beach, CA, USA), a closed polyacrylamide gel electrophoresis platform. Serum samples were stored at −20 °C; kits were equilibrated at room temperature for 2 h before use. Photoactive-dye loading gel was mixed with serum, loaded into polyacrylamide tubes, and polymerized under white light for 35 min. Following electrophoresis, gel columns were scanned and analyzed with Lipoprint System Research Software (ImageSXM v1.82, Quantimetrix). LDL subfractions were classified as large LDL (buoyant) and small LDL (dense); HDL subfractions as large, intermediate, and small HDL.

### 2.5. Statistical Analysis

The distribution of continuous variables was assessed prior to analysis using visual inspection and statistical criteria. Accordingly, appropriate parametric or non-parametric tests were applied. Statistical analyses were performed using SPSS version 26.0 (IBM Corp., Armonk, NY, USA). Continuous variables are reported as mean ± standard deviation (SD); categorical variables as frequencies and percentages. Group comparisons used independent Student’s *t*-test or Mann–Whitney U test for continuous variables, and Chi-square (χ^2^) for categorical variables. Hardy–Weinberg equilibrium (HWE) was assessed by χ^2^ in the control group. Genotype-stratified lipid comparisons used Kruskal–Wallis H test (three groups) or Mann–Whitney U test (TT carriers vs. non-carriers). Multivariable logistic regression was performed to identify independent predictors of CAD, adjusting for CT genotype, TT genotype, age, sex, BMI, HDL-C, diabetes mellitus, and smoking (reference genotype: CC). Model calibration was assessed by the Hosmer-Lemeshow test; model performance reported as Nagelkerke R^2^. Multivariable linear regression models evaluated the association of TT genotype (dominant model: TT vs. CC+CT) with each lipid subfraction, adjusting for age, sex, and BMI; model fit is reported as F-statistic, *p*-value, R^2^, and adjusted R^2^. Due to the small TT sample (n = 6–9 in subfraction analyses), results are considered exploratory and were not Bonferroni-corrected for multiple comparisons; all reported *p*-values are uncorrected two-tailed, with significance set at *p* < 0.05.

### 2.6. Sample Size and Power Analysis

A priori sample size calculation was performed using G*Power (version 3.1.9.7; Faul et al., 2007) [24]. Calculations were based on a medium effect size, consistent with previous recommendations for genetic association studies in complex diseases such as coronary artery disease.

Two primary analyses were considered. First, for the comparison of genotype distribution between CAD patients and controls using the chi-square test (codominant model, 2 × 3 contingency table, df = 2), assuming an effect size of w = 0.30, α = 0.05, and power (1 − β) = 0.80, the minimum required sample size was 111 participants. Under the dominant model (2 × 2 contingency table, df = 1), the required sample size was 88 participants.

Second, for comparisons of lipid subfraction levels across genotype groups using one-way ANOVA, assuming an effect size of f = 0.30, α = 0.05, and power (1 − β) = 0.80, the required sample size was 158 participants for the codominant model and 176 participants for the dominant model. The most conservative required sample size was therefore 176 participants.

The final study cohort consisted of 264 participants (139 CAD patients and 125 controls), exceeding the most conservative required sample size by approximately 50%. Accordingly, the study was considered adequately powered for both the primary genetic association analyses and the lipid subfraction comparisons [24].

### 2.7. In Silico Functional Annotation

In silico functional annotation of the CXCL12 rs1801157 polymorphism was performed to evaluate its potential biological and regulatory significance. Publicly available bioinformatics databases, including the Genotype-Tissue Expression (GTEx) project and RegulomeDB, were used for this purpose.

Expression quantitative trait locus (eQTL) analysis was conducted using the GTEx database to investigate potential effects of rs1801157 on CXCL12 gene expression across multiple human tissues. No statistically significant eQTL associations were identified for rs1801157 in the available datasets.

To assess the regulatory potential of the variant, RegulomeDB was used to evaluate chromatin state annotations, transcription factor binding potential, and chromatin accessibility. The rs1801157 variant demonstrated a RegulomeDB rank score of 5, suggesting limited but potential regulatory functionality. The variant was also found to overlap with regions of chromatin accessibility and enhancer-associated chromatin states in multiple tissues, including immune-related cell types [25,26,27].

## 3. Results

### 3.1. Study Population and Baseline Characteristics

A total of 264 participants were enrolled: 139 CAD patients and 125 healthy controls. CAD patients had significantly higher body weight (78.97 ± 14.82 vs. 73.18 ± 13.87 kg; *p* = 0.007) and BMI (28.58 ± 5.92 vs. 26.40 ± 4.94 kg/m^2^; *p* = 0.008). Critically, serum HDL-C was markedly lower in patients (38.70 ± 8.52 vs. 44.04 ± 11.82 mg/dL; *p* = 0.001), reinforcing its established role as a discriminating cardiovascular biomarker. No significant differences were observed in total cholesterol, LDL-C, VLDL-C, or triglycerides between groups (all *p* > 0.05; Table 1).

### 3.2. C-X-C Motif Chemokine Ligand 12 (CXCL12) rs1801157 Genotype Distribution and CAD Risk

Genotype and allele frequencies are presented in Table 2. In CAD patients, CC, CT, and TT genotype frequencies were 51.1%, 38.1%, and 10.8%, respectively; controls showed 55.2%, 35.2%, and 9.6%. No statistically significant difference in overall genotype distribution was observed (χ^2^ = 0.459, df = 2, *p* = 0.796), and neither individual genotype comparisons nor allele frequencies reached significance (all *p* > 0.05). Hardy–Weinberg equilibrium was confirmed in the control group (*p* > 0.05).

Multivariable logistic regression, adjusting for age, sex, BMI, HDL-C, diabetes mellitus, and smoking (reference: CC genotype), confirmed that neither CT (OR = 1.396, 95% CI: 0.626–3.114; *p* = 0.409) nor TT genotype (OR = 1.458, 95% CI: 0.214–9.951; *p* = 0.694) was an independent predictor of CAD risk. The model demonstrated good calibration (Hosmer-Lemeshow χ^2^ = 10.2, df = 8, *p* = 0.136) and explained 33.1% of variance in CAD outcome (Nagelkerke R^2^ = 0.331). Three clinical variables emerged as independent predictors: HDL-C was a significant independent protective factor (OR = 0.952, 95% CI: 0.910–0.996; *p* = 0.029), indicating that each 1 mg/dL increment in HDL-C reduces CAD odds by approximately 4.8%. However, diabetes mellitus was not significantly associated with CAD in the adjusted model (OR = 1.651, 95% CI: 0.853–3.173, *p* = 0.132). Active smoking was modestly associated with CAD (OR = 1.853, 95% CI: 1.002–3.424, *p* = 0.049). Age and sex showed borderline associations (both *p* = 0.053), while BMI was not independently associated with CAD (*p* = 0.431).

### 3.3. Lipoprotein Subfraction Analysis: Patient–Control Differences and Genotype Effects

Detailed subfraction data are presented in Table 3. Compared to controls, CAD patients exhibited a significantly more atherogenic lipoprotein profile: small LDL particles were elevated (6.23 ± 7.52 vs. 3.65 ± 6.80 mg/dL; *p* = 0.041), while total HDL-C (*p* = 0.001) and small HDL subfractions (6.36 ± 3.26 vs. 7.86 ± 3.48 mg/dL; *p* = 0.012) were significantly reduced. Large HDL and intermediate HDL did not differ significantly between groups (both *p* > 0.05).

When lipoprotein subfractions were stratified by C-X-C motif chemokine ligand 12 *(CXCL12)* rs1801157 genotype, TT homozygous carriers (n = 6–9) displayed a markedly superior antiatherogenic profile relative to CC+CT non-carriers. Specifically, TT carriers had significantly higher large HDL (15.83 ± 2.32 vs. 12.65 ± 6.83 mg/dL; *p* = 0.018) and intermediate HDL (25.67 ± 1.51 vs. 21.26 ± 5.21 mg/dL; *p* < 0.001), alongside substantially lower small LDL particles (1.86 ± 1.35 vs. 5.20 ± 7.44 mg/dL; *p* < 0.001). No significant genotype-related differences were found in total HDL-C or small HDL (both *p* > 0.05), underscoring the selectivity of the rs1801157 effect for functionally relevant subfraction species. Given the limited TT sample size, these subfraction findings are considered exploratory.

### 3.4. Multivariable Linear Regression: TT Genotype as Independent Modulator of Lipoprotein Subfractions

To determine whether the TT genotype’s association with lipoprotein subfractions was independent of classical confounders, multivariable linear regression models were constructed with TT genotype as the primary predictor (dominant model: TT vs. CC+CT) and age, sex, and BMI as covariates (Table 4). The TT genotype was a significant independent predictor of three key subfractions: increased large HDL (B = 3.18, β = 0.142, t = 2.50, *p* = 0.018; model: F(4,125) = 3.12, *p* = 0.018, R^2^ = 0.091), increased intermediate HDL (B = 4.41, β = 0.241, t = 4.50, *p* < 0.001; model: F(4,125) = 5.84, *p* < 0.001, R^2^ = 0.157), and decreased small LDL (B = −3.34, β = −0.198, t = −3.84, *p* < 0.001; model: F(4,122) = 4.47, *p* = 0.002, R^2^ = 0.128). These effects persisted after full covariate adjustment. In contrast, total HDL-C (F(4,149) = 1.43, *p* = 0.226, R^2^ = 0.037) and small HDL (F(4,125) = 0.075, *p* = 0.990, R^2^ = 0.002) models were non-significant, indicating a selective, subfraction-specific effect of the rs1801157 variant on lipoprotein quality rather than overall lipid quantity.

These findings carry mechanistic significance: large and intermediate HDL particles are the most functionally active subfractions in RCT, while small dense LDL is the most atherogenic LDL class. The concurrent independent upregulation of cardioprotective HDL subfractions and downregulation of atherogenic small LDL in TT carriers suggests a coherent antiatherogenic lipid phenotype potentially mediated through CXCL12/CXCR4-ABCA1 signaling pathways, although dedicated mechanistic studies are required to confirm this.

### 3.5. In Silico Functional Annotation

The rs1801157 polymorphism is located in the 3′ untranslated region of the CXCL12 gene. GTEx database analysis did not identify significant expression quantitative trait locus (eQTL) associations for rs1801157 across available tissues. RegulomeDB annotation suggested limited regulatory potential for this variant, with a rank score of 5. The variant was found to overlap with enhancer-associated chromatin states and regions of open chromatin accessibility in multiple tissues, including immune-related cell types [25,26,27].

### 3.6. Summary of Principal Findings

(i) *CXCL12* rs1801157 is not an independent predictor of CAD in this Turkish cohort. (ii) HDL-C (OR = 0.952; *p* = 0.029) and smoking (OR = 1.853; *p* = 0.049) are the principal independent determinants of CAD risk. (iii) TT homozygous carriers show significantly elevated large HDL (*p* = 0.018) and intermediate HDL (*p* < 0.001) and reduced small LDL (*p* < 0.001)—a coherent antiatherogenic subfraction phenotype. (iv) These subfraction effects are independent of age, sex, and BMI (linear regression R^2^ = 0.091–0.157 for significant models), suggesting the rs1801157 variant modulates lipoprotein quality rather than quantity.

## 4. Discussion

Coronary artery disease (CAD) is a complex multifactorial disorder characterized by progressive atherosclerosis driven by the interplay of chronic inflammation, endothelial dysfunction, immune activation, and disturbances in lipid metabolism [20,28,29]. In the present study, we investigated the potential role of the *CXCL12* rs1801157 C>T polymorphism in CAD susceptibility and its association with detailed lipoprotein subfraction profiles in a Turkish cohort. The main findings of our study are twofold: (i) rs1801157 was not independently associated with CAD risk, and (ii) TT homozygosity was associated with a more favorable lipoprotein subfraction pattern, characterized by increased large and intermediate HDL particles and reduced small dense LDL particles.

C-X-C motif chemokine ligand 12 *(CXCL12)* (stromal cell-derived factor-1) is a chemokine that plays a central role in leukocyte trafficking and atherogenesis through interaction with its receptor C-X-C motif chemokine receptor 4 (*CXCR4*). Activation of *CXCL12/CXCR4* signaling has been implicated in the recruitment of inflammatory cells into the vascular wall and in the progression of atherosclerotic lesions via downstream pathways including MAPK and PI3K signaling [9,11,12]. Beyond its inflammatory effects, emerging evidence suggests that *CXCL12* may also influence lipid metabolism and cholesterol homeostasis. In experimental models, *CXCL12/CXCR4* signaling has been shown to modulate ATP-binding cassette transporter A1 (*ABCA1)* expression through intracellular pathways involving GSK3β and β-catenin, potentially affecting cholesterol efflux and HDL biogenesis [17,18,20]. 

The rs1801157 C>T polymorphism is located in the 3′ untranslated region of the *CXCL12* gene and has been suggested to influence gene expression regulation, although findings across studies remain inconsistent. It is therefore plausible that this variant may exert subtle regulatory effects on *CXCL12* expression or mRNA stability, rather than inducing large functional changes [30,31]. In this context, the observed association between TT homozygosity and a more favorable HDL and LDL subfraction profile may reflect a modest modulation of lipid handling pathways rather than a direct or strong transcriptional effect [32].

The biological plausibility of the observed findings is partially suggested by in silico functional annotation of the CXCL12 rs1801157 polymorphism. Although no significant eQTL associations were identified in GTEx datasets, RegulomeDB analysis suggested that rs1801157 may be located within regulatory genomic regions characterized by enhancer-associated chromatin marks and chromatin accessibility. These results indicate a possible but limited regulatory potential for this variant at the transcriptional level. Given the established role of CXCL12 in immune cell trafficking, inflammatory signaling, and tissue homeostasis, even modest regulatory effects may be biologically relevant in the context of complex multifactorial diseases such as atherosclerosis [25,26,27].

Genome-wide association studies (GWASs) have consistently demonstrated that coronary artery disease (CAD) is a highly polygenic disorder involving hundreds of susceptibility loci distributed across the genome. Large-scale meta-analyses, particularly those conducted by the CARDIoGRAMplusC4D Consortium, which included over one million individuals, have identified more than 250 CAD-associated loci and confirmed the involvement of multiple biological pathways related to lipid metabolism, vascular integrity, and inflammatory signaling. These studies have established the 10q11 chromosomal region as one of the relevant susceptibility loci for CAD, thereby implicating the *CXCL12* genomic region in atherosclerotic disease biology [22,33,34]. However, despite the robust identification of the 10q11 locus at the genome-wide level, subsequent analyses of specific single nucleotide polymorphisms within this region, including *CXCL12* rs1801157, have yielded inconsistent results across different populations. In particular, large GWAS meta-analyses have not demonstrated a strong or reproducible association between rs1801157 and CAD risk, suggesting that its effect size is likely modest or dependent on population-specific genetic architecture [35]. This is consistent with the present study, in which rs1801157 was not independently associated with CAD susceptibility in a Turkish cohort. Recent multi-ancestry genomic studies further support the biological relevance of the *CXCL12* locus in coronary vascular biology and cardiovascular phenotypes, suggesting that the contribution of this region to CAD may involve complex regulatory and developmental mechanisms beyond single-variant disease associations [36].

Importantly, GWAS are primarily designed to detect associations with binary disease outcomes and may not capture intermediate metabolic phenotypes such as lipoprotein subfraction distribution or functional HDL characteristics [22,37]. In contrast, our findings suggest that rs1801157 is associated with significant alterations in HDL and LDL subfraction profiles without translating into a measurable effect on CAD risk. This discrepancy may indicate that *CXCL12* variation may exert its biological influence through subtle regulatory mechanisms affecting lipid metabolism rather than directly determining clinical CAD endpoints [38,39].

HDL functionality is now recognized as being more closely related to particle size and composition than to total HDL-C concentration [16]. Low HDL-C levels remain a clinical challenge and continue to be associated with increased cardiovascular risk, although their interpretation as a therapeutic target remains complex [40]. Clinical studies have also suggested that HDL-C levels may still be associated with endothelial function and cardiovascular outcomes, particularly in interventional settings [41]. Large HDL particles are enriched in apolipoprotein A-I and antioxidant enzymes such as paraoxonase-1 (PON1), and they exhibit greater capacity to mediate reverse cholesterol transport (RCT) via scavenger receptor class B type I (SR-BI)-dependent hepatic uptake [16,20]. In contrast, small HDL particles are less efficient in cholesterol esterification and hepatic delivery [16]. Moreover, experimental and clinical studies have shown that HDL particle size positively correlates with cholesterol efflux capacity, a key antiatherogenic function of HDL [15,16]. In parallel, small dense LDL particles are more atherogenic due to their increased susceptibility to oxidation, prolonged plasma half-life, and enhanced arterial wall penetration [42]. Therefore, the shift toward larger HDL and reduced small LDL observed in TT carriers suggests a more favorable lipoprotein functionality profile, potentially reflecting improved cholesterol trafficking dynamics.

Despite these favorable subfraction changes, rs1801157 was not independently associated with CAD risk in our cohort. This dissociation between lipid subfraction phenotype and clinical disease outcome is not unexpected. Atherosclerosis is a polygenic and multifactorial process in which environmental factors such as smoking, systemic inflammation, insulin resistance, and metabolic comorbidities often exert stronger effects than individual genetic variants [10]. In our multivariate analysis, HDL-C levels and smoking status remained the principal independent determinants of CAD risk, consistent with established cardiovascular risk models.

Moreover, the lack of association between rs1801157 and CAD risk in our study aligns with several previous reports in different populations [15,22,41]. The modest effect size of single polymorphisms, combined with ethnic variability, linkage disequilibrium patterns, and gene–environment interactions, may contribute to inconsistent findings across studies. In contrast, our results suggest that rs1801157 may act more as a modifier of lipoprotein quality rather than a direct determinant of CAD susceptibility. This may partly explain why favorable subfraction changes do not necessarily translate into measurable differences in clinical CAD outcomes.

A key observation of this study is the apparent dissociation between genetically associated lipoprotein subfraction changes and CAD risk. Although TT homozygosity was linked to a more anti-atherogenic lipid profile, this did not confer a statistically significant reduction in CAD risk. This may indicate that modulation of lipoprotein quality alone is insufficient to overcome the cumulative burden of traditional cardiovascular risk factors. Alternatively, longer-term longitudinal exposure or interaction with other genetic variants may be required for such metabolic differences to translate into clinically detectable effects on atherosclerotic disease progression.

When compared with studies in other populations, our findings suggest potential ethnic and population-specific effects of rs1801157. Several studies in European and Asian cohorts have reported no significant associations between this polymorphism and lipid parameters or CAD risk [22,34,35]. In contrast, our results demonstrate a clear association with HDL and LDL subfraction distribution in a Turkish cohort. These discrepancies may reflect differences in genetic background, linkage disequilibrium patterns, dietary habits, and environmental exposures, all of which can modulate chemokine-related metabolic pathways.

In addition, the high prevalence of cardiometabolic and stroke-related risk factors in geographically and metabolically similar populations such as Türkiye and Albania, including dyslipidemia, hypertension, obesity, smoking, insulin resistance, and sedentary lifestyle, may further influence lipoprotein metabolism and atherosclerotic susceptibility [43,44]. Recent epidemiological studies from these populations have highlighted that adverse lipid profiles, particularly elevated LDL/HDL and TG/HDL ratios, are strongly associated with ischemic stroke and cardiovascular disease risk [43]. Furthermore, growing evidence indicates that antiatherogenic lipoprotein subfractions, especially large HDL particles, exert protective effects through reverse cholesterol transport, antioxidant activity, and anti-inflammatory mechanisms, whereas small dense LDL particles display enhanced atherogenicity due to their greater susceptibility to oxidation and endothelial penetration [45,46]. Therefore, population-specific metabolic and environmental factors may contribute to the distinct lipoprotein subfraction patterns observed in our Turkish cohort and may partly explain the stronger association detected between rs1801157 and antiatherogenic lipoprotein profiles compared with other ethnic groups.

Beyond lipid metabolism–related mechanisms, recent evidence suggests that malignancies, particularly gynecological cancers, share several common molecular and pathophysiological mechanisms with atherosclerotic diseases, including chronic inflammation, oxidative stress, endothelial dysfunction, and dysregulated lipid metabolism [47]. Epidemiological studies have demonstrated that patients with ovarian, endometrial, and cervical cancers frequently exhibit an increased prevalence of cardiovascular risk factors such as obesity, hypertension, insulin resistance, and dyslipidemia, all of which may contribute to accelerated atherosclerotic progression [48]. In addition, cancer-related inflammatory mediators, including chemokines and cytokines, may promote vascular injury and plaque instability through persistent activation of immune and endothelial cells [49]. Emerging evidence also indicates that altered lipoprotein subfraction profiles, particularly reduced large HDL particles and increased small dense LDL particles, are associated with both tumor progression and cardiovascular risk, suggesting a possible mechanistic link between carcinogenesis and atherogenesis [50]. Furthermore, anticancer treatments such as chemotherapy, radiotherapy, and endocrine therapy may exacerbate endothelial dysfunction and metabolic disturbances, thereby increasing long-term cardiovascular morbidity in gynecological cancer survivors [51]. Therefore, the overlap between malignancy-related inflammatory pathways and atherosclerotic mechanisms may partly explain the coexistence of cardiovascular disease and gynecological tumors in susceptible populations.

Overall, these findings support the concept that inflammatory and metabolic pathways involved in atherosclerosis may also contribute to broader systemic disease processes. In this context, the observed association between *CXCL12* rs1801157 and antiatherogenic lipoprotein subfractions may reflect subtle regulatory effects on lipid homeostasis and vascular biology rather than direct effects on CAD susceptibility. Although no independent association with CAD risk was identified, our results suggest that genetic variation within the *CXCL12* pathway may influence intermediate metabolic phenotypes related to cardiovascular risk. Further large-scale and longitudinal studies are needed to better clarify the mechanistic role of *CXCL12* signaling in lipoprotein metabolism and cardiometabolic disease progression.

### Limitations

The primary limitation of this study is the small number of TT homozygous individuals (n = 6–9 in subfraction analyses), which reduces statistical power and limits the robustness of subgroup analyses. Therefore, the observed lipoprotein subfraction findings should be interpreted as exploratory and hypothesis-generating rather than definitive. The cross-sectional design of the study further precludes any causal inference regarding the relationship between *CXCL12* rs1801157 and lipid metabolism or coronary artery disease.

Despite these limitations, the consistent directionality of the observed associations across multiple lipoprotein subfractions supports the potential biological relevance of the findings. Functional validation studies assessing *CXCL12* expression levels in TT carriers, cholesterol efflux capacity, and replication in larger and ethnically diverse cohorts are required to confirm and extend these observations.

## 5. Conclusions

This study provides evidence, for the first time, that the C-X-C motif chemokine ligand 12 (*CXCL12*) rs1801157 polymorphism is associated with HDL subfraction distribution in a Turkish population. Although the variant was not independently associated with coronary artery disease susceptibility, TT homozygosity was linked to a more favorable anti-atherogenic lipoprotein profile, characterized by increased large and intermediate HDL subfractions and decreased small dense LDL particles.

These associations remained significant after adjustment for classical cardiovascular risk factors, suggesting that *CXCL12* genetic variation may contribute to inter-individual differences in lipoprotein quality through modulation of lipid metabolic pathways. However, further large-scale, prospective, and mechanistic studies are necessary to clarify the clinical relevance of these findings and their potential role in cardiovascular risk stratification.

## Figures and Tables

**Table 1:** Baseline Demographic and Lipid Characteristics of CAD Patients and Controls.

Variable	Patients (*n* = 139)	Controls (*n* = 125)	*p*-Value
**Demographic characteristics**			
Age (years)	61.77 ± 8.69	59.53 ± 9.96	0.109
Weight (kg)	78.97 ± 14.82	73.18 ± 13.87	**0.007**
Body mass index (BMI, kg/m^2^)	28.58 ± 5.92	26.40 ± 4.94	**0.008**
Male sex, n (%)	68 (48.9%)	57 (45.6%)	0.321
Diabetes mellitus, n (%)	63 (52.6%)	51 (47.4%)	0.131
Current smoking, n (%)	72 (61.4%)	57 (39.6%)	**0.049**
**Standard lipid profile**			
Total cholesterol (mg/dL)	185.80 ± 46.88	191.94 ± 37.17	0.370
LDL-C (mg/dL)	118.44 ± 39.63	122.29 ± 34.09	0.518
HDL-C (mg/dL)	38.70 ± 8.52	44.04 ± 11.82	**0.001**
VLDL-C (mg/dL)	29.44 ± 10.54	30.68 ± 17.72	0.576
Triglycerides (mg/dL)	148.94 ± 56.23	153.35 ± 89.69	0.702

Values are Mean ± SD or n (%). BMI: body mass index; LDL-C: low-density lipoprotein cholesterol; HDL-C: high-density lipoprotein cholesterol; VLDL-C: very low-density lipoprotein cholesterol. Comparisons by independent Student’s *t*-test or Chi-square (χ^2^) test. Bold *p*-values indicate statistical significance (*p* < 0.05).

**Table 2:** *CXCL12* rs1801157 Genotype/Allele Distribution and Multivariable Logistic Regression for CAD Risk.

	Patients (*n* = 139)	Controls (*n* = 125)	*p*-Value	OR	95% CI
**Genotype Distribution—Chi-Square Test**					
CC, n (%)	71 (51.1%)	69 (55.2%)	0.503	0.847	0.522–1.376
CT, n (%)	53 (38.1%)	44 (35.2%)	0.622	1.135	0.687–1.774
TT, n (%)	15 (10.8%)	12 (9.6%)	0.588	1.254	0.553–2.842
Overall genotype: χ^2^ = 0.459, df = 2, *p* = 0.796|C allele: 70.1% vs.)|T allele: 29.9% vs. 27.2% (*p* = 0.580)|HWE *p* > 0.05					
**Multivariable logistic regression †—dependent variable: CAD (yes/no)**					
**Variable**	**B**	**Wald χ^2^**	***p*** **-value**	**OR**	**95% CI**
CT genotype	0.333	0.680	0.409	1.396	0.626–3.114
TT genotype	0.378	0.154	0.694	1.458	0.214–9.951
HDL-C (mg/dL)	−0.049	4.794	**0.029**	**0.952**	**0.910–0.996**
Diabetes mellitus	0.502	2.267	0.132	1.651	0.853–3.173
Smoking	0.617	3.870	**0.049**	**1.853**	**1.002–3.424**
Age (years)	0.048	3.752	0.053	1.049	0.999–1.101
Sex (male)	0.796	3.757	0.053	2.216	0.988–4.967
BMI (kg/m^2^)	0.036	0.619	0.431	1.037	0.948–1.134
Model fit: −2LL = 219.4; Cox & Snell R^2^ = 0.247; Nagelkerke R^2^ = 0.331; Hosmer-Lemeshow χ^2^ = 10.2, df = 8, *p* = 0.136. Overall classification accuracy: 73.1%.					

† Multivariable logistic regression adjusted for age, sex, BMI, HDL-C, diabetes mellitus, and smoking. Reference genotype: CC. OR: odds ratio; CI: confidence interval; B: regression coefficient; HWE: Hardy–Weinberg equilibrium; HL: Hosmer-Lemeshow. Highlighted cells indicate *p* < 0.05.

**Table 3:** Lipoprotein Subfraction Levels in CAD Patients vs. Controls and According to *CXCL12* rs1801157 TT Carrier Status.

Lipoprotein Subfraction	Patients (*n* = 139)	Controls (*n* = 125)	*p* (Group)	TT Carriers † (*n* = 6–9)	*p* (TT)
**LDL subfractions**					
Large LDL (mg/dL)	55.81 ± 19.42	53.76 ± 15.18	0.501	61.86 ± 7.11	0.281
Small LDL (mg/dL)	6.23 ± 7.52	3.65 ± 6.80	**0.041**	**1.86 ± 1.35 ↓↓**	**<0.001**
**HDL subfractions**					
Total HDL-C (mg/dL)	38.70 ± 8.52	44.04 ± 11.82	**0.001**	41.56 ± 6.73	0.803
Large HDL (mg/dL)	12.34 ± 6.78	13.37 ± 6.65	0.390	**15.83 ± 2.32 ↑↑**	**0.018**
Intermediate HDL (mg/dL)	21.41 ± 5.31	21.53 ± 5.05	0.900	**25.67 ± 1.51 ↑↑**	**<0.001**
Small HDL (mg/dL)	6.36 ± 3.26	7.86 ± 3.48	**0.012**	6.50 ± 2.95	0.680

Values are Mean ± SD. Patient vs. control comparisons by independent Student’s *t*-test. TT carrier vs. non-carrier comparisons by Mann–Whitney U test († indicates n = 6–9 for TT carriers). ↑↑ significantly higher; ↓↓ significantly lower in TT carriers vs. CC+CT. Highlighted cells indicate *p* < 0.05.

**Table 4:** Multivariable Linear Regression: TT Genotype as Independent Predictor of Lipoprotein Subfractions.

Dependent Variable	B	SE	β	t	*p*-Value	Model Fit
Large HDL (mg/dL)	3.18	1.27	0.142	2.50	**0.018**	F(4,125) = 3.12, *p* = 0.018; R^2^ = 0.091; Adj. R^2^ = 0.062
Intermediate HDL (mg/dL)	4.41	0.98	0.241	4.50	**<0.001**	F(4,125) = 5.84, *p* < 0.001; R^2^ = 0.157; Adj. R^2^ = 0.130
Small LDL (mg/dL)	−3.34	0.87	−0.198	−3.84	**<0.001**	F(4,122) = 4.47, *p* = 0.002; R^2^ = 0.128; Adj. R^2^ = 0.099
Small HDL (mg/dL)	−0.583	1.47	−0.023	−0.40	0.699	F(4,125) = 0.075, *p* = 0.990; R^2^ = 0.002
Total HDL-C (mg/dL)	0.62	1.13	0.031	0.55	0.803	F(4,149) = 1.43, *p* = 0.226; R^2^ = 0.037

Dominant model: TT vs. CC+CT. Covariates: age, sex, BMI. B: unstandardized coefficient; SE: standard error; β: standardized coefficient; t: t-statistic. R^2^: coefficient of determination; Adj. R^2^: adjusted R^2^. All *p*-values are uncorrected two-tailed. Highlighted cells indicate *p* < 0.05.

## Data Availability

Dataset available on request from the authors.

## Abbreviations

The following abbreviations are used in this manuscript:

BMI	Body mass index
*CXCL12*	The C-X-C motif chemokine ligand 12
CXCR4	The C-X-C motif chemokine receptor 4
HDL	High-density lipoprotein
HDL-C	HDL cholesterol
LDL	Low-density lipoprotein
LDL-C	LDL cholesterol
MAPK	Mitogen-activated protein kinase
PI3K	phosphoinositide 3-kinase
RCT	Reverse cholesterol transport
SNP	Single nucleotide polymorphism
SYNTAX	Synergy Between PCI With Taxus and Cardiac Surgery score
